# Enhanced Topological Network Efficiency in Preschool Autism Spectrum Disorder: A Diffusion Tensor Imaging Study

**DOI:** 10.3389/fpsyt.2018.00278

**Published:** 2018-06-27

**Authors:** Bin Qin, Longlun Wang, Yun Zhang, Jinhua Cai, Jie Chen, Tingyu Li

**Affiliations:** ^1^Department of Radiology, Children's Hospital of Chongqing Medical University, Chongqing, China; ^2^Children Nutrition Research Center, Children's Hospital of Chongqing Medical University, Ministry of Education Key Laboratory of Child Development and Disorders, China International Science and Technology Cooperation Base of Child Development and Critical Disorders, Chongqing Key Laboratory of Translational Medical Research in Cognitive Development and Learning and Memory Disorders, Chongqing, China

**Keywords:** autism spectrum disorder (ASD), DTI, graph theory, network efficiency, preschool children

## Abstract

**Background:** The functional mechanism behind autism spectrum disorder (ASD) is not clear, but it is related to a brain connectivity disorder. Previous studies have found that functional brain connectivity of ASD is linked to both increased connections and weakened connections, and the inconsistencies in functional brain connectivity may be related to age. The functional connectivity in adolescents and adults with ASD is generally less than in age-matched controls; functional connectivity in younger children with the disorder appears to be higher. As the basis of the functional network, the structural network is less studied. This study intends to further study the pathogenesis of ASD by analyzing the white matter network of ASD preschool children.

**Materials and Methods:** In this study, Diffusion Tensor Imaging (DTI) was used to scan preschool children (aged 2–6 years, 39 children with ASD, 19 children as controls), and graph theory was used for analysis.

**Result:** Enhanced topological network efficiency was found in the preschool children with ASD. A higher nodal efficiency was found in the left precuneus, thalamus, and bilateral superior parietal cortex, and the nodal efficiency of the left precuneus was positively associated with the severity of ASD.

**Conclusion:** Our research shows the white matter network efficiency of preschoolers with ASD. It supports the theory of excessive early brain growth in ASD, and it shows left brain lateralization. It opens the way for new research perspectives of children with ASD.

## Introduction

Autism spectrum disorder (ASD) refers to a range of conditions characterized by abnormalities in social communication and development and by repetitive behaviors and restricted interests; it is a set of prevalent disorders (1, 2). ASD is believed to result from an interaction between genetic, developmental, and environmental factors, and it has a current global prevalence of 1.5% in developed countries (3). In the last decade, ASD occurrence has increased and currently reaches about 1 per 68 children. Therefore, early diagnosis and effective treatment have become extremely important public health concerns.

The neuropathological abnormalities that define ASD are still lacking, but it is obvious that ASD is correlated with abnormalities in neuroconnectivity (4, 5). However, some results of neuroconnectivity studies are inconsistent, leaving the exact role of neuroconnectivity abnormalities in question (6). For example, the resting-state connection with the cingulate gyrus has been shown to be enhanced in some cortical areas and decreased in others in individuals with ASD (7). In addition, results on the relationship between neuroconnectivity and ASD features are inconsistent. For example, the poor social ability of ASD patients is associated with both the decrease (8) and increase (9) of resting-state connections.

Although it is difficult to reconstruct brain tissue structure, the ongoing development of neuroimaging has opened the possibility to conduct some in-depth studies of the brain tissue of ASD patients. Unlike the case in many other neurodevelopmental disorders, there is much evidence that early brain development in children with ASD is accelerated (10–12). This early swift growth has been thought to affect the interhemispheric and cortico-cortical connections (13). The process of myelination has not been completed before the age of 2 years (14). Children older than 6 years of age have already begun to receive education in primary school, and different levels of education will inevitably lead to different brain development, so the early years, including 2–6 years, are an important period of neural network formation, which is crucial to early intervention and brain development (15).

Diffusion tensor imaging (DTI) is a type of MRI technology that can detect the macroscopic WM pathways and the microstructure of WM by measuring the diffusion of water in the brain (16). DTI tractography reflects the WM fiber of the brain region and is helpful to understanding the structural connection (17, 18). Functional magnetic resonance imaging (fMRI) is another type of MRI technology that measures brain activity by detecting changes associated with blood flow (19). When an area of the brain is in use, blood flow to that region also increases.

Graph theory is the study of graphs, which are mathematical structures used to model pairwise relations between objects. It is especially suitable for the comprehensive study of the characteristics of the whole brain network (20–22). Some previous studies using DTI or fMRI with graph theory in ASD patients have been published (22–24), but there are no studies using graph theory to analyze DTI images of children aged 2–6 years with ASD.

In this study, we hypothesize that ASD has an anomaly in the topology of the structural network. We collected brain structure data (i) from 39 outpatient children with ASD who attend special education institutions and (ii) from 19 normal control children without ASD. Then, we used DTI technology to establish the structural network. We defined the nodes as 90 brain regions and we defined the edges as the mean fractional anisotropy (FA) value of the paired regions. Finally, we analyzed the topological properties with graph theory and nonparametric tests, and we made group comparisons using the topological data.

## Materials and methods

### Participants

The parents of each child signed consent, and the protocol was approved by the Children Hospital of Chongqing Medical University Research Ethics Committee. All of the participants were from the Children Hospital of Chongqing Medical University. Of these, 39 were children with ASD (age: 2.89 ± 0.97 years) and 19 were healthy controls (age: 3.15 ± 1.12 years). A clinician gave the diagnosis of ASD according to the DSM-IV-TR criteria (25). The severity of ASD was assessed according to the Childhood Autism Rating Scale (CARS). All of the children had reports of normal neurologic examinations in their medical records. The criteria for exclusion of healthy children included known diseases of the nervous system and a history of systemic or neurodevelopment disease or psychosis. Children with ASD did not receive any central nervous system-active medications before the MRI studies. Table 1 provides the subjects' age and gender and the clinical assessment scores.

**Table 1:** Age, gender, and the clinical scales of the two groups of children.

**Variables**	**ASD (*n* = 39)**	**Controls (*n* = 19)**	***p*-value**
Average age in years (mean ± SD)	2.89 ± 0.97	3.15 ± 1.12	0.36^a^
Gender (M/F)	32/7	13/6	0.24^b^
CARS (mean ± *SD*)	33.67 ± 1.49		

a *The difference of the two groups was tested by two-sample t-test*.

b *The difference of the two groups was tested by chi-square test*.

### MR data acquisition

Each subject's MR data were collected by a 3Tesla Philips Achieva MR-scanner with an 8-channel head coil.

The T1-weighted images (T1WIs) were acquired using a sagittal three-dimensional SPGR sequence. The parameters of the sequence were the following: repetition time (TR), 7.7 ms; echo time (TE), 3.8 ms; flip angle, 8°; field of view (FOV), 256 × 256; voxel size, 1 mm × 1 mm × 1 mm; slice thickness, 1 mm; total time: 155 min.

The diffusion tensor images (DTIs) were acquired using an axial single-shot echo-planar imaging (EPI) sequence. The parameters of the sequence were as follows: TR, 9,155 ms; TE, 65 ms; flip angle, 90°; FOV, 230 × 230; voxel size, 1.8 × 1.8 × 2 mm; slice thickness, 2 mm; *b*-value, 1,000; total time, 386 min.

### Network construction by graph theory

The brain network can be described by nodes and edges, where nodes and edges can be defined in many ways. We used the following methods to define nodes and edges (26).

#### Network node definition

First, we need to match the space of the DTI with the space of T1 (27). Therefore, each T1WI is first registered with the B0 image of the DTI through a linear transformation. Then, by applying affine transformation, the co-registered structure image is mapped to the Montreal Neurosciences Institute (MNI)T1 template, and a series of nonlinear distortion is used to simulate the affine transformation.

The obtained conversion parameters were retrieved and applied to the automated anatomical labeling (AAL) regions from the MNI space to the DTI space (28). Statistical Parametric Mapping (SPM8, http://www.fil.ion.ucl.ac.uk/spm) was applied to carry out the preprocessing, through which 90 cortical and subcortical regions (45 for each hemisphere) have been obtained.

#### Network edge definition

Due to the eddy current distortion, DTIs were geometrically corrected for their stretches and shears; then we used B0 image affine transformation on the record to reduce mild head movement. The linear least-squares fitting method was used to estimate the diffusion tensor model. We used the Fiber Assignment by Continuous Tracking (FACT) algorithm to track whole brain fiber for each subject in native diffusion space using the Diffusion Toolkit. All of the trajectories in the data are calculated using the seeding element of FA > 0.2. Either when the FA-value of a voxel is <0.2 or the angle between the current and the former path segment is over 45°, the path tracing would suspend (29).

To determine the edge, two regions are thought to be connected by the edges if at least one fiber appears between them. We computed the mean FA-value of the connecting fiber between two regions for each side of the network. The FA-value plays an important role in evaluating the integrity of WM fiber (30). Several studies have shown that the mean FA-value of a brain network is more readily available than that of a fiber bundle of brain networks, because the mean FA can reflect local damage of the brain.

### Analysis of network topology attributes

#### Small-world attributes of the network

Small-world properties were initially proposed by Watts and Strogatz (31). We focus on the small-world properties of the structure network of the brain in this study. The weighted clustering coefficient of a node *i*, ${\text{C}}_{\text{i}}^{w}$, was used to measure how close the node *i*'s neighbors are to be a clique. It is expressed as follows:

$$\begin{array}{l} C_{i}^{w}=\frac{1}{k_{i}\left(k_{i}-1\right)}\underset{j,h\in N}{\sum }{\left(w_{ij}w_{ih}w_{jh}\right)}^{1/3}, \end{array}$$

where *k*_*i*_ is the number of edges connecting to node *i* and *w*_*ij*_is the weight between node *i* and node *j* in this network. The overall weighted clustering coefficient, ${}_{{\text{C}}^{\text{w}}}$, represents the average of $C_{i}^{w}$ across all of the nodes; its expression is$C^{w}=\frac{1}{N}\sum_{i\in N}C_{i}^{w}$, where *N* is the number of nodes. The weighted clustering coefficient represents the local interconnection degree or the partiality of the network.

The path length between nodes *i* and *j* is defined as the sum of the lengths along this path. In this study, the weighted structure network is calculated by calculating the reciprocal of edge weights,1/*w*_*ij*_. The shortest path length, *L*_*ij*_, between the nodes *i* and *j* is the shortest path between the two nodes, and the number of edges is the shortest path length between the two nodes. The weighted characteristic shortest path length *L*^*w*^ of a network was measured by the “harmonic mean” length between pairs. In other words, we have computed the reciprocal of all nodes to solve the problem of the network components that may be disconnected, as shown below:

$$\begin{array}{l} L^{w}=\frac{N\left(N-1\right)}{\overset{N}{\underset{i=1}{\sum }}\overset{N}{\underset{j\neq i}{\sum }}1/L_{ij}}, \end{array}$$

where *N* is the number of nodes. The shortest path length reflects the optimal path of information transferred from one node to another in the network, and it plays an important role in the global information transmission within the network (32).

If there is a similar path length, but with a higher clustering coefficient than a random network, the real world will be regarded as a small world, that is, $\gamma =C^{w}/C_{random}^{w}>1$, $\lambda =L^{w}/L_{random}^{w}\approx 1$ (31). The $C_{random}^{w}$ is the average of the weighted clustering coefficient. The $L_{random}^{w}$ is the mean weighted characteristic shortest path length of matched random networks. We can combine two measurements as scalar quantization measures, small-worldness, _σ = γ/λ_, which is typically >1 in the case of small-world organization (33).

#### Efficiency of the network

The global efficiency, *E*_*global*_, is defined by the inverse of the harmonic mean of the minimum path length between each pair of nodes. It is expressed as$E_{global}=\frac{1}{N\left(N-1\right)}\underset{i\neq j\in N}{\sum }\frac{1}{L_{i,j}}$, where *N* is the number of nodes of the network and *E*_*global*_is the standard to measure the global transmission capacity of information (32).

Local efficiency, *E*_*local*_, is defined as the average of the local efficiency of each node. It is expressed as$E_{local}=\frac{1}{N}\underset{i\in G}{\sum }E_{global}\left(G_{i}\right)$, where *E*_*global*_(*G*_*i*_) is the global efficiency of the neighborhood subgraph *G*_*i*_ of the node *i* and *E*_*local*_shows how each subgraph exchanges information when the index node is eliminated (32).

#### Characteristics of the nodes

In order to evaluate the network topological properties of the local brain regions, three kinds of methods were applied: nodal strength $k_{i}^{w}$, nodal efficiency $e_{i}^{w}$, and betweenness $b_{i}^{w}$.

The degree of a node *i* was defined as the number of edges that are directly connected to the node *i*. The degree is a simple measure of network connections between nodes and other nodes. The total weighted connection strength, ${}_{{\text{S}}^{\text{w}}}$, was computed as the mean of all nodes *N* in a network (34) as follows:$S^{w}=\frac{1}{N}\underset{i\in G}{\sum }k_{i}^{w}$.

The nodal efficiency of node *i*, $e_{i}^{w}$, is computed as $e_{i}^{w}=\frac{1}{N-1}\underset{i\neq j\in G}{\sum }\frac{1}{L_{i,j}}$. The nodal efficiency represents the ability to transfer information from one point to another in a network (20).

The betweenness of node I, $B_{i}^{w}$, is calculated as the fraction of all shortest paths through a node's network. We calculated the normalized betweenness as $b_{i}^{w}{=}^{B_{i}^{w}}{/}_{\langle B_{i}^{w}\rangle }$, where $\langle B_{i}^{w}\rangle$ is the average betweenness of the network in this study. Betweenness captures the effect of nodes on the flow of information between all other nodes in the network (26).

It is noteworthy that the centrality measurement of the nodes defined above reflects the importance of network nodes from different aspects. For example, a high-degree node can be regarded as the center of information integration; the high-efficient nodes are related to the information flow; and those nodes with high betweenness may be used as a network hub.

### Statistical analysis

#### Differences in the network metrics

In consideration of the small sample size of the current study, graph metrics of the brain ($C^{w},L^{w},\lambda ,\gamma ,\delta ,S^{w},E_{local},E_{global},k_{i}^{w},e_{i}^{w},b_{i}^{w}$) were compared between the ASD group and the control group by using a nonparametric permutation test. Briefly, we first calculated the intergroup difference of each graph metric. We then randomly assigned each participant to one of the two groups with the same size as the original ASD and control groups. This randomization procedure was repeated for 10000 permutations, which generated a null permutation distribution. For each permutation, the new intergroup difference was calculated. We then assigned a *P*-value to the intergroup difference by computing the proportion of differences exceeding the null distribution values. For the metrics of$C^{w},L^{w},\lambda ,\gamma ,\delta ,S^{w},E_{local},E_{global}$, *P*-values below the statistical threshold value of *P* = 0.05 were considered significant. A false discovery rate (FDR) with *Q* < 0.05 was employed to correct multiple comparisons for nodal metrices ($k_{i}^{w},e_{i}^{w},b_{i}^{w}$).

#### Relationships between the network measures metrics and CARS

Once the significant differences in network metrics between the ASD and control groups were found, an exploratory investigation was conducted to evaluate the linear relationship between those metrics and the CARS scores of the ASD group using Pearson correlation analysis. A statistical result of *P* < 0.05 was considered to be significant.

## Results

### Economic small-world brain structural networks

**Figure 1:**
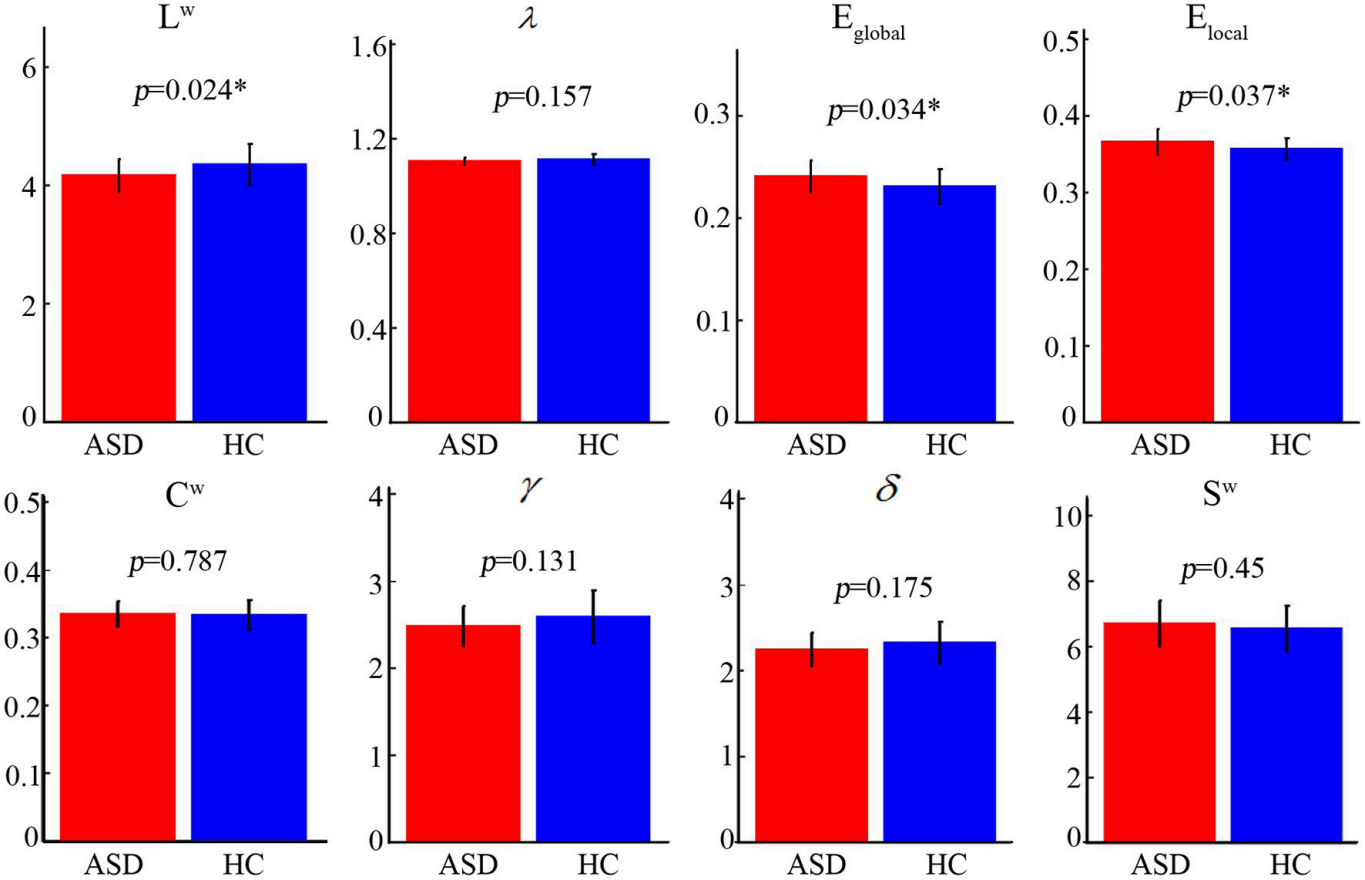
Different topological properties of the two group's structural connectivity networks. The asterisks represent a statistically significant difference (nonparametric permutation test, *P* < 0.05). The characteristics of the shortest path length (*L*^*w*^), the global efficiency (*E*_*global*_), and the local efficiency (*E*_*local*_) of the two groups were significantly different. ASD, autism spectrum disorder; HC, healthy control.

The normalized path length of the ASD group and the control group is approximately 1, the normalized clustering coefficient is >1 (Figure 1), and it can be considered as a small-world topology. These results show the economic small-world topology in the two groups.

### Altered overall topological properties in ASD

Statistical analysis revealed that the ASD group showed significantly lower L^w^ (ASD [mean ± SD]: 4.17 ± 0.28; HC [mean ± SD]: 4.35 ± 0.35; *P* = 0.024) and higher E_global_ (ASD [mean ± SD]: 0.24 ± 0.02; HC [mean ± SD]: 0.23 ± 0.02; *P* = 0.034) and E_local_ (ASD [mean ± SD]: 0.37 ± 0.02; HC [mean ± SD]: 0.36 ± 0.01; *P* = 0.037) (Figure 1). No significant differences (*P* > 0.05) were found in*C*^*w*^, γ, λ, σ, and*S*^*w*^.

### ASD-related alteration in nodal centralities

**Figure 2:**
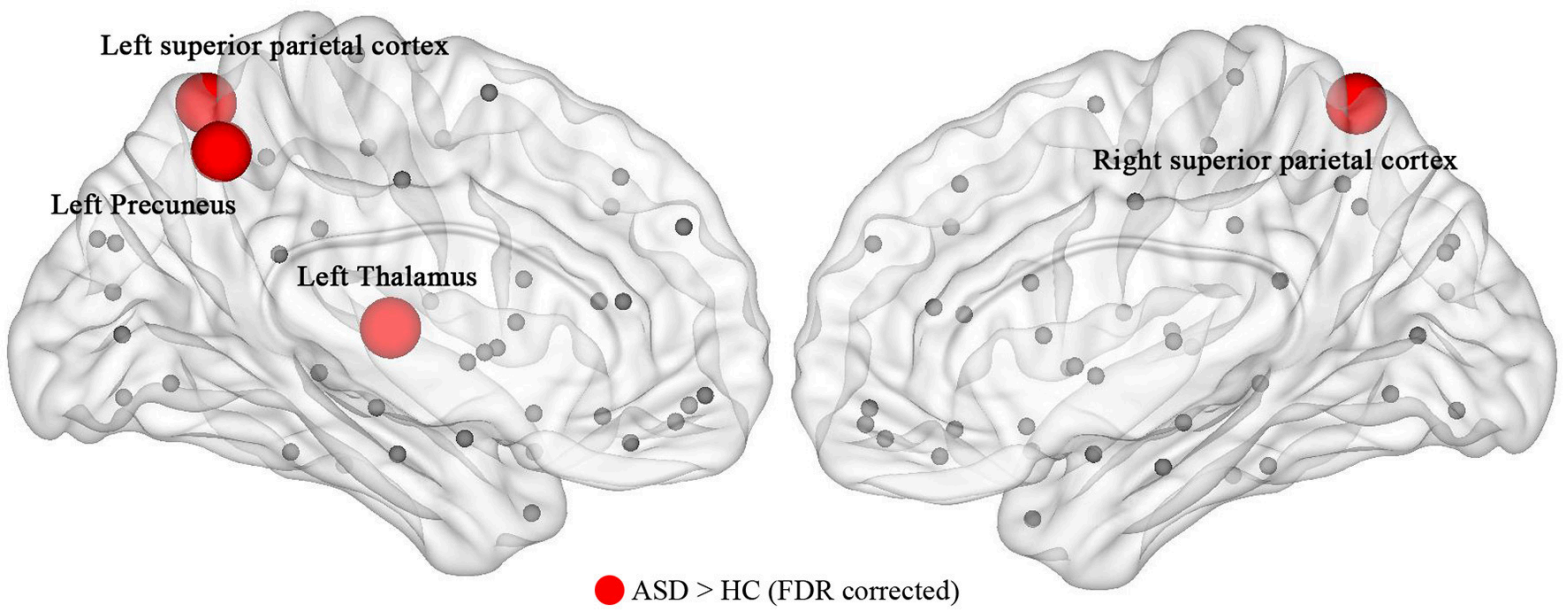
The nodal efficiency of the structural network showed a significant (*P* ≤ 0.05, FDR-corrected) alteration in children with ASD. The result was visualized by using the BrainNet viewer (NKLCNL, Beijing Normal University). Red balls represent the areas of increased nodal efficiency in ASD children. ASD, autism spectrum disorder; HC, healthy control.

The ASD group exhibited significantly higher nodal efficiency (FDR-corrected, *Q* < 0.05) in the left precuneus, thalamus, and bilateral superior parietal cortex, compared with controls (Figure 2). There was no significant difference in nodal strength and betweenness between the two groups.

### Relationship between the network metrics and the CARS

**Figure 3:**
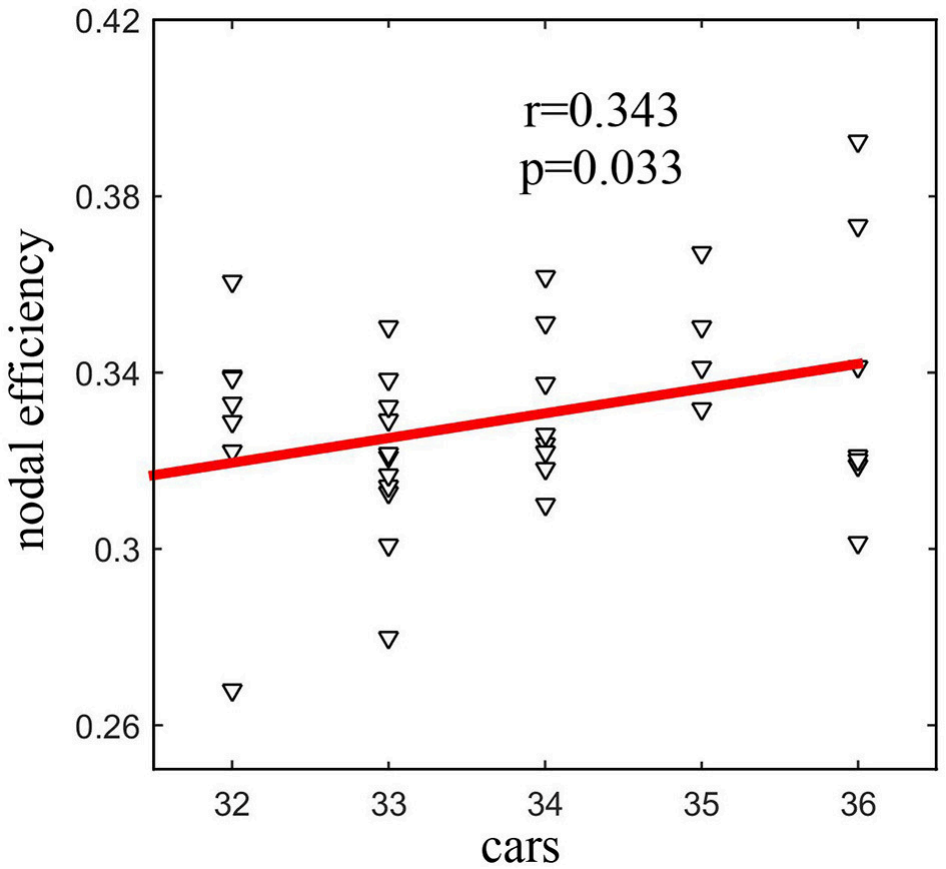
Statistically significant positive correlation (*r* = 0.343, *P* = 0.033) between the nodal efficiency of the left precuneus and the CARS score in ASD.

Seven network metrics in total (*L*^*w*^, *E*_*local*_, *E*_*global*_, and $e_{i}^{w}$ of four nodes) showed a difference between the groups. Among those metrics, there was a statistically significant positive correlation (*r* = 0.343, *P* = 0.033) between nodal efficiency of the left precuneus and the CARS score in ASD patients (Figure 3).

## Discussion

This study provides evidence for the altered structural network in ASD children aged 2–6 years. The topology of the structural network was found to be disrupted in the children with ASD, with the decreased values of *L*^*w*^ and increased values of *E*_*global*_ and *E*_*local*_. The results showed that there is an interference between the local specialization and global integration of ASD children. Additionally, ASD children revealed increased nodal efficiency of some brain areas, including the left precuneus, thalamus, and bilateral superior parietal cortex. The nodal efficiency of the left precuneus is related to CARS, which represents the severity of ASD. These results provide a structural basis for an easier understanding of neuropathological abnormalities in ASD children.

The human brain can be conceptualized as a network. The small-world model is well suited for complex brain dynamics because it supports efficient information segregation and integration with low energy and wiring costs (e.g., a high rate of information transmission) (31). There are some studies that show the human brain network is characterized by a small-world organization (20, 21, 35). We found that both the ASD group and the control group showed topological small-world network properties in this study.

Although both groups have small-world properties, there are still some topological differences. The ASD group exhibited a significantly decreased *L*^*w*^ and an increased *E*_*local*_ and *E*_*global*_ in their brain network. This has led some to suggest that there is an increase in both short-range and long-range brain connectivity in ASD; it also means that the brain structures of children with ASD aged 2–6 are overconnected. The overconnectivity may be a result of early brain overgrowth in ASD children (36–39). Several scholars have dissected the brain of recently deceased autistic persons and they found that the frontal lobe shows an increase in WM fiber tracts. It may be that this structural anomaly leads to abnormal brain networks (39).

The ASD-related increase in nodal centrality was mainly found in the left precuneus, thalamus, and bilateral superior parietal cortex. We found a statistically significant positive correlation between the nodal efficiency of the left precuneus and the CARS score in autism. The precuneus is part of the default module network (DMN), which has been implicated in many psychiatric disorders, including ASD and ADHD. The DMN is a core brain system for processing information about the “self” and “other” and has emerged as a key system underlying social dysfunction in ASD. Other studies reported abnormalities in the left precuneus in ASD compared with normal controls. Mak-Fan et al. found a thicker cortex in the left precuneus gyrus (40). Jiang et al. studied the data of 539 ASD (aged 17.01 ± 8.37) and 573 healthy controls (aged 17.08 ± 7.72), they found that individuals with ASD show increased ReHo levels in the left precuneus with age, while typical controls show decreased ReHo levels with age (41). More importantly, in this study, the nodal efficiency of the left precuneus increased with an increased severity of autism. Therefore, the left precuneus may be a target region for future developmental studies of ASD.

The thalamus, a subcortical–cortical relay that had been mostly ignored in many MRI studies, demonstrated the most striking of the FC abnormalities. Recently, Schuetze et al. (42) studied the date of Autism Brain Imaging Date Exchange (373 male participants aged 7–35 years with ASD and 384 typically developing), and found that ASD males reveal smaller age-related increases in thalamic local functional connectivity density, which were associated with symptoms of autism. Tomasi et al. (43) found the similar phenomenon. In our study, we found that the node efficiency of the left thalamus was increased, so we should pay more attention to the role of the thalamus in ASD research.

The superior parietal cortex is part of the dorsal attention network (DAN). The DAN is concerned with top-down, goal-directed attention. Pruett found that children with ASD made more saccades and had slower reaction times during visual orienting (44). This means the orienting system, of which the DAN constitutes a significant part, is impaired in children with ASD. Farrant found that children (aged 7–13 years) with ASD show hyper-connectivity in the DAN and that adults (aged 18–39 years) with ASD show hypoconnectivity in the DAN (45). In our study, we found the nodal efficiency of the superior parietal cortex increased in children (aged 2–6 years) with ASD. This also confirms previous research results from a new perspective.

Lateralization of brain structure and brain function during brain development is normal. The left-lateralized hubs include language regions (e.g., the Wernicke Area and the Broca Area) and the areas from the DMN (posterior cingulate cortex (PCC), precuneus, medial prefrontal cortex (mPFC), temporoparietal junction (TPJ), and hippocampus), which processes information about the “self” and “other.” The right-lateralized hubs include regions from the attention control network (e.g., frontal eye fields and the lateral intraparietal sulcus) (46). Atypical lateralization in the brain structure or function is relevant to neuropsychiatric disorders, such as ASD (47, 48). In this study, nodes with higher node efficiency are located on the left side of the brain, indicating that the excessive enhancement of the left network efficiency of children with ASD may be one of the reasons for language dysfunction and social dysfunction.

Several limitations of this study need to be addressed. First, the definition of nodes is 90 partitions of the automatic anatomic labeling (AAL) template whose boundaries are not accurate enough, so the nodes need more accurate partition templates to be defined. Second, in the current study a longitudinal aspect of ASD lacks, and the age was relatively limited (2–6 years), so a follow-up study is needed to increase the longitudinality of the study. Third, this study only collected the data of the structure and failed to combine the structure with the functional network, so a follow-up study is needed to increase the amount of data on the brain function data to conduct a comparison study.

## Conclusion

Our research studied the WM brain network of preschool (aged 2–6 years) children with ASD. The local and global network efficiency of the WM brain network of children with ASD is enhanced, which supports the overgrowth theory of the early brain. At the same time, we found an increased nodal efficiency of the left precuneus, thalamus, and bilateral superior parietal cortex; these brain areas are rarely mentioned in other studies. We also found the nodal efficiency of the left precuneus is positively correlated with the severity of ASD, which has also been rarely mentioned before. These findings may provide a new direction for further study of the mechanism behind ASD.

## Ethics statement

This study was carried out in accordance with the recommendations of together for children, Children Hospital of Chongqing Medical University Research Ethics Committee. The protocol was approved by the Children Hospital of Chongqing Medical University Research Ethics Committee. All subjects gave written informed consent in accordance with the Declaration of Helsinki.

## Author contributions

BQ: carry out the implementation of the project, participate in the selection of the subjects, the scanning of MR and the processing of the data in the later stage; LW and YZ: MR scan parameter setting and scan the participants; JinC: pointing to image scanning sequences and applications in autism; JieC: point to specific topic design; TL: design, implement, and check the project progress.

### Conflict of interest statement

The authors declare that the research was conducted in the absence of any commercial or financial relationships that could be construed as a potential conflict of interest.

## References

[1] McPartland J Volkmar FR. Autism and related disorders. Handb Clin Neurol. (2012) 106:407–18. 10.1016/B978-0-444-52002-9.00023-1 22608634 PMC3848246

[2] Roehr B. American Psychiatric Association explains DSM-5. BMJ (2013) 346:f3591. 10.1136/bmj.f3591 23744600

[3] Baxter AJ Brugha TS Erskine HE Scheurer RW Vos T Scott JG. The epidemiology and global burden of autism spectrum disorders. Psychol Med. (2015) 45:601–13. 10.1017/S003329171400172X 25108395

[4] Castelli F Frith C Happe F Frith U. Autism, Asperger syndrome and brain mechanisms for the attribution of mental states to animated shapes. Brain (2002) 125(Pt 8):1839–49. 10.1093/brain/awf189 12135974

[5] Just MA Cherkassky VL Keller TA Minshew NJ. Cortical activation and synchronization during sentence comprehension in high-functioning autism: evidence of underconnectivity. Brain (2004) 127(Pt 8):1811–21. 10.1093/brain/awh199 15215213

[6] Vissers ME Cohen MX Geurts HM. Brain connectivity and high functioning autism: a promising path of research that needs refined models, methodological convergence, and stronger behavioral links. Neurosci Biobehav Rev. (2012) 36:604–25. 10.1016/j.neubiorev.2011.09.003 21963441

[7] Monk CS Peltier SJ Wiggins JL Weng SJ Carrasco M Risi S. Abnormalities of intrinsic functional connectivity in autism spectrum disorders. Neuroimage (2009) 47:764–72. 10.1016/j.neuroimage.2009.04.069 19409498 PMC2731579

[8] Weng SJ Wiggins JL Peltier SJ Carrasco M Risi S Lord C. Alterations of resting state functional connectivity in the default network in adolescents with autism spectrum disorders. Brain Res. (2010) 1313:202–14. 10.1016/j.brainres.2009.11.057 20004180 PMC2818723

[9] Anderson JS Nielsen JA Froehlich AL DuBray MB Druzgal TJ Cariello AN. Functional connectivity magnetic resonance imaging classification of autism. Brain (2011) 134(Pt 12):3742–54. 10.1093/brain/awr263 22006979 PMC3235557

[10] Courchesne E. Brain development in autism: early overgrowth followed by premature arrest of growth. Ment Retard Dev Disabil Res Rev. (2004) 10:106–11. 10.1002/mrdd.20020 15362165

[11] Chawarska K Campbell D Chen L Shic F Klin A Chang J. Early generalized overgrowth in boys with autism. Arch Gen Psychiatry (2011) 68:1021–31. 10.1001/archgenpsychiatry.2011.106 21969460 PMC4878118

[12] Hazlett HC Poe MD Gerig G Styner M Chappell C Smith RG. Early brain overgrowth in autism associated with an increase in cortical surface area before age 2 years. Arch Gen Psychiatry (2011) 68:467–76. 10.1001/archgenpsychiatry.2011.39 21536976 PMC3315057

[13] Herbert MR Ziegler DA Makris N Filipek PA Kemper TL Normandin JJ Jr. Localization of white matter volume increase in autism and developmental language disorder. Ann Neurol. (2004) 55:530–40. 10.1002/ana.20032 15048892

[14] Miyamachi K Miyasaka K Abe H. [The MR evaluation of normal children and disorders of neuronal migration and myelination]. No To Shinkei (1990) 42:183–8. 2357420

[15] Sowell ER Bookheimer SY. Promise for finding brain biomarkers among infants at high familial risk for developing autism spectrum disorders. Am J Psychiatry (2012) 169:551–3. 10.1176/appi.ajp.2012.12030397 22684588

[16] Basser PJ Mattiello J LeBihan D. MR diffusion tensor spectroscopy and imaging. Biophys J. (1994) 66:259–67. 10.1016/S0006-3495(94)80775-1 8130344 PMC1275686

[17] Basser PJ Pajevic S Pierpaoli C Duda J Aldroubi A. *In vivo* fiber tractography using DT-MRI data. Magn Reson Med. (2000) 44:625–32. 10.1002/1522-2594(200010)44:4<625::AID-MRM17>3.0.CO;2-O 11025519

[18] Shu N Liu Y Li K Duan Y Wang J Yu C. Diffusion tensor tractography reveals disrupted topological efficiency in white matter structural networks in multiple sclerosis. Cereb Cortex (2011) 21:2565–77. 10.1093/cercor/bhr039 21467209

[19] Ogawa S Lee TM Kay AR Tank DW. Brain magnetic resonance imaging with contrast dependent on blood oxygenation. Proc Natl Acad Sci USA. (1990) 87:9868–72. 10.1073/pnas.87.24.9868 2124706 PMC55275

[20] Bullmore E Sporns O. Complex brain networks: graph theoretical analysis of structural and functional systems. Nat Rev Neurosci. (2009) 10:186–98. 10.1038/nrn2575 19190637

[21] Micheloyannis S. Graph-based network analysis in schizophrenia. World J Psychiatry (2012) 2:1–12. 10.5498/wjp.v2.i1.1 24175163 PMC3782171

[22] Ibrahim GM Morgan BR Vogan VM Leung RC Anagnostou E Taylor MJ. Mapping the network of neuropsychological impairment in children with autism spectrum disorder: a graph theoretical analysis. J Autism Dev Disord. (2016) 46:3770–7. 10.1007/s10803-016-2929-8 27696182

[23] Rudie JD Brown JA Beck-Pancer D Hernandez LM Dennis EL Thompson PM. Altered functional and structural brain network organization in autism. Neuroimage Clin. (2012) 2:79–94. 10.1016/j.nicl.2012.11.006 24179761 PMC3777708

[24] Hernandez LM Rudie JD Green SA Bookheimer S Dapretto M. Neural signatures of autism spectrum disorders: insights into brain network dynamics. Neuropsychopharmacology (2015) 40:171–89. 10.1038/npp.2014.172 25011468 PMC4262896

[25] APA (2000). Diagnostic and Statistical Manual of Mental Disorders, 4th edn. Washington, DC: American Psychiatric Association.

[26] Long Z Duan X Xie B Du H Li R Xu Q. Altered brain structural connectivity in post-traumatic stress disorder: a diffusion tensor imaging tractography study. J Affect Disord. (2013) 150:798–806. 10.1016/j.jad.2013.03.004 23684515

[27] Gong G He Y Concha L Lebel C Gross DW Evans AC. Mapping anatomical connectivity patterns of human cerebral cortex using *in vivo* diffusion tensor imaging tractography. Cereb Cortex (2009) 19, 524–36. 10.1093/cercor/bhn102 18567609 PMC2722790

[28] Tzourio-Mazoyer N Landeau B Papathanassiou D Crivello F Etard O Delcroix N. Automated anatomical labeling of activations in SPM using a macroscopic anatomical parcellation of the MNI MRI single-subject brain. Neuroimage (2002) 15, 273–89. 10.1006/nimg.2001.0978 11771995

[29] Zhu L Guo G. An improved fiber tracking algorithm based on fiber assignment using the continuous tracking algorithm and two-tensor model. Neural Regen Res. (2012) 7:1667–1674. 10.3969/j.issn.1673-5374.2012.21.010 25657708 PMC4308771

[30] Tuch DS Wedeen VJ Dale AM George JS Belliveau JW. Conductivity tensor mapping of the human brain using diffusion tensor MRI. Proc Natl Acad Sci USA. (2001) 98:11697–701. 10.1073/pnas.171473898 11573005 PMC58792

[31] Watts DJ Strogatz SH. Collective dynamics of 'small-world' networks. Nature (1998) 393:440–2. 10.1038/30918 9623998

[32] Latora V Marchiori M. Efficient behavior of small-world networks. Phys Rev Lett (2001) 87, 198701. 10.1103/PhysRevLett.87.198701 11690461

[33] Humphries MD Gurney K Prescott TJ. The brainstem reticular formation is a small-world, not scale-free, network. Proc Biol Sci. (2006) 273:503–11. 10.1098/rspb.2005.3354 16615219 PMC1560205

[34] Zhang Z Liao W Chen H Mantini D Ding JR Xu Q. Altered functional-structural coupling of large-scale brain networks in idiopathic generalized epilepsy. Brain (2011) 134(Pt 10):2912–28. 10.1093/brain/awr223 21975588

[35] Minati L Grisoli M Seth AK Critchley HD. Decision-making under risk: a graph-based network analysis using functional MRI. Neuroimage (2012) 60:2191–2205. 10.1016/j.neuroimage.2012.02.048 22387471

[36] Lainhart JE Piven J Wzorek M Landa R Santangelo SL Coon H. Macrocephaly in children and adults with autism. J Am Acad Child Adolesc Psychiatry (1997) 36:282–90. 10.1097/00004583-199702000-00019 9031582

[37] Courchesne E Carper R Akshoomoff N. Evidence of brain overgrowth in the first year of life in autism. JAMA (2003) 290:337–44. 10.1001/jama.290.3.337 12865374

[38] Courchesne E Campbell K Solso S. Brain growth across the life span in autism: age-specific changes in anatomical pathology. Brain Res. (2011) 1380:138–45. 10.1016/j.brainres.2010.09.101 20920490 PMC4500507

[39] Courchesne E Mouton PR Calhoun ME Semendeferi K Ahrens-Barbeau C Hallet MJ. Neuron number and size in prefrontal cortex of children with autism. JAMA (2011) 306:2001–10. 10.1001/jama.2011.1638 22068992

[40] Mak-Fan KM Taylor MJ Roberts W Lerch JP. Measures of cortical grey matter structure and development in children with autism spectrum disorder. J Autism Dev Disord. (2012) 42:419–27. 10.1007/s10803-011-1261-6 21556969

[41] Jiang L Hou XH Yang N Yang Z Zuo XN. Examination of local functional homogeneity in autism. Biomed Res Int. (2015) 2015:174371. 10.1155/2015/174371 26180782 PMC4477064

[42] Schuetze M Park MT Cho IY MacMaster FP Chakravarty MM Bray SL. Morphological alterations in the thalamus, striatum, and pallidum in autism spectrum disorder. Neuropsychopharmacology (2016) 41:2627–37. 10.1038/npp.2016.64 27125303 PMC5026732

[43] Tomasi D Volkow ND. (2017) Reduced local and increased long-range functional connectivity of the thalamus in autism spectrum disorder. Cereb Cortex. [Epub ahead of print]. 10.1093/cercor/bhx340 29300843 PMC6319176

[44] Pruett J. R. Jr LaMacchia A Hoertel S Squire E McVey K Todd RD. Social and non-social cueing of visuospatial attention in autism and typical development. J Autism Dev Disord. (2011) 41:715–31. 10.1007/s10803-010-1090-z 20809377 PMC3660145

[45] Farrant K Uddin LQ. Atypical developmental of dorsal and ventral attention networks in autism. Dev Sci. (2016) 19:550–63. 10.1111/desc.12359 26613549

[46] Nielsen JA Zielinski BA Ferguson MA Lainhart JE Anderson JS. An evaluation of the left-brain vs. right-brain hypothesis with resting state functional connectivity magnetic resonance imaging. PLoS ONE (2013) 8:e71275. 10.1371/journal.pone.0071275 23967180 PMC3743825

[47] Fletcher PT Whitaker RT Tao R DuBray MB Froehlich A Ravichandran C. E. Microstructural connectivity of the arcuate fasciculus in adolescents with high-functioning autism. Neuroimage (2010) 51:1117–25. 10.1016/j.neuroimage.2010.01.083 20132894 PMC2966943

[48] Lange N Dubray MB Lee JE Froimowitz MP Froehlich A Adluru N. Atypical diffusion tensor hemispheric asymmetry in autism. Autism Res. (2010) 3:350–8. 10.1002/aur.162 21182212 PMC3215255

