# Corrigendum: Enhanced Topological Network Efficiency in Preschool Autism Spectrum Disorder: A Diffusion Tensor Imaging Study

**DOI:** 10.3389/fpsyt.2019.00068

**Published:** 2019-02-19

**Authors:** Bin Qin, Longlun Wang, Yun Zhang, Jinhua Cai, Jie Chen, Tingyu Li

**Affiliations:** ^1^Department of Radiology, Children's Hospital of Chongqing Medical University, Chongqing, China; ^2^Children Nutrition Research Center, Children's Hospital of Chongqing Medical University, Ministry of Education Key Laboratory of Child Development and Disorders, China International Science and Technology Cooperation Base of Child Development and Critical Disorders, Chongqing Key Laboratory of Translational Medical Research in Cognitive Development and Learning and Memory Disorders, Chongqing, China

**Keywords:** autism spectrum disorder (ASD), DTI, graph theory, network efficiency, preschool children

In the original article, we neglected to include the funder the “National Natural Science Foundation of China, 81471518 and 81771223” to Tingyu Li.

The authors apologize for this error and state that this does not change the scientific conclusions of the article in any way. The original article has been updated.

